# High neutrophils and low lymphocytes percentages in bronchoalveolar lavage fluid are prognostic factors of higher in-hospital mortality in diffuse alveolar hemorrhage

**DOI:** 10.1186/s12890-021-01660-x

**Published:** 2021-09-09

**Authors:** Kensuke Kanaoka, Seigo Minami, Shoichi Ihara, Kiyoshi Komuta

**Affiliations:** Department of Respiratory Medicine, Osaka Police Hospital, 10-31, Kitayama-cho, Tennoji-ku, Osaka-City, Osaka 543-0035 Japan

**Keywords:** Diffuse alveolar hemorrhage, Bronchoalveolar lavage, Neutrophils, Lymphocytes, Estimated glomerular filtration rate, In-hospital mortality, Antithrombotic therapy

## Abstract

**Background:**

Diffuse alveolar hemorrhage (DAH) is a syndrome resulting from bleeding in the microcirculation of the lung, with a poor prognosis. The study aim was to identify prognostic factors of DAH, especially bronchoalveolar lavage fluids (BALF) cell pattern.

**Methods:**

We conducted a single-center retrospective cohort study of patients diagnosed as having DAH and hospitalized at our hospital between October 2008 and July 2020. We performed univariate logistic regressions to identify variables associated with in-hospital death.

**Results:**

Sixty-eight patients were included in our analysis. In-hospital mortality was 26.5%. Variables associated with in-hospital death were neutrophils percentage in BALF ≥ 44.5% [Odds Ratio (OR) 16.0, 95% confidence interval (CI) 4.33–58.9)], lymphocytes percentage in BALF < 14% (OR 7.44, 95% CI 2.11–26.2), idiopathic DAH (OR 0.31, 95% CI 0.10–0.95), oxygen flow ≥ 4L/min (OR 3.90, 95% CI 1.20–12.6), and estimated glomerular filtration rate < 60 mL/min (OR 5.00, 95%CI 1.29–19.4).

**Conclusions:**

High neutrophils and low lymphocytes percentages in BALF were associated with poor prognosis.

**Supplementary Information:**

The online version contains supplementary material available at 10.1186/s12890-021-01660-x.

## Background

Diffuse alveolar hemorrhage (DAH) is a clinicopathological syndrome that describes the accumulation of intra-alveolar red blood cells originating from the alveolar capillaries [1]. DAH can induce severe respiratory failure. In-hospital mortality has ranged from 15 to 51% in previous studies [2–5]. Additionally, some clinical characteristics and biological parameters have been proven to be prognostic factors of DAH [2, 3, 5].

Bronchoalveolar lavage (BAL) is an essential DAH diagnostic method [6]. The BAL fluid (BALF) of DAH presents visually hemorrhagic and cytologically hemosiderin-laden macrophages [7]. However, to the best of our knowledge, the association between the BALF cell pattern and prognosis has never been investigated in DAH.

Antithrombotic therapy (AT) is known to be one of the causes of DAH, and other etiologies can induce DAH during AT [4, 8]. Only two studies have investigated the association between AT and prognosis in DAH [2, 9]. In those studies, the mortality was not significantly different between DAH that occurred during AT (DAH-AT) and DAH that occurred without AT (DAH-NAT) [2, 9]. On the other hand, one study showed that prognosis was better for DAH-AT with no cause other than AT (simple DAH-AT) than for DAH-AT with causes other than AT (complicated DAH-AT) and DAH-NAT [9]. However, considering the current trend toward more patients receiving AT, the prognostic influence of AT in DAH remains insufficient [10]. Consequently, it is important to investigate the relationship between AT and prognosis and characterize the differences in prognoses among complicated DAH-AT and simple DAH-AT.

Supplemental Fig. 1 Receiver operating characteristic curves for predicting in-hospital death for the factors oxygen flow (A), neutrophils percentage (B), and lymphocytes percentage (C) in bronchoalveolar lavage fluid. AUC: area under curve; BALF: bronchoalveolar lavage fluid.

**Fig. 1:**
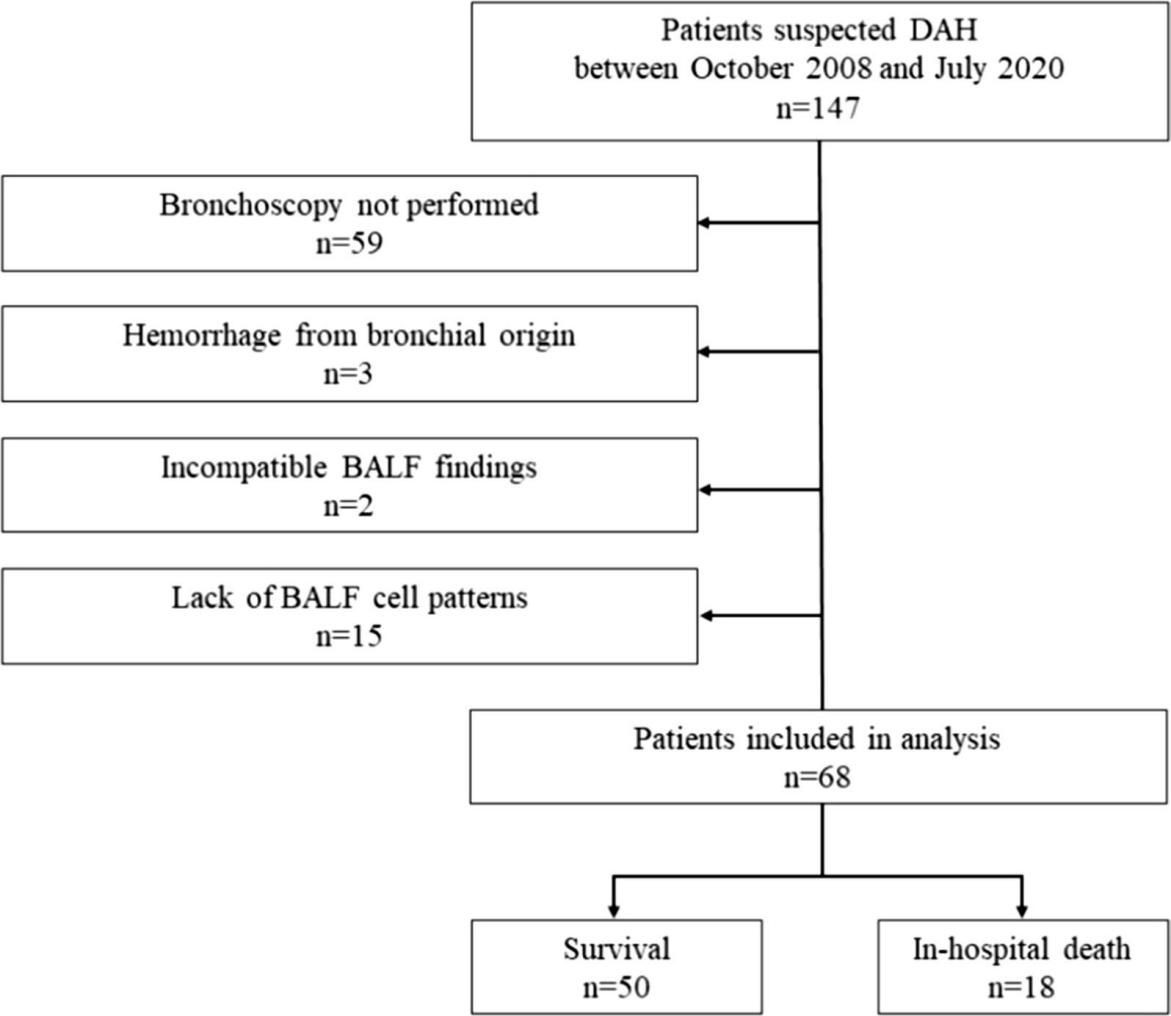
Flow chart of patients suspected of having diffuse alveolar hemorrhage (DAH)

In this study, we aimed to reveal the prognostic factor in DAH, especially BALF cell pattern.

## Methods

### Study design

We conducted a retrospective cohort study in our hospital. The study was approved by the ethics committee of Osaka Police Hospital (Osaka, Japan) and the medical records of patients admitted between October 2008 and July 2020 were reviewed. If a patient had been admitted due to DAH two or more times during this period, only the first admission was included in the analysis. In this study, DAH was defined by the following criteria: (1) Clinical and radiological presentation that was compatible with a diagnosis of DAH, and (2) BALF that was macroscopically bloody or cytologically contained hemosiderin-burden macrophages. The exclusion criteria were as follows: (1) Hemorrhage of bronchial origin, (2) BALF findings incompatible with DAH, and (3) Lack of BALF cell-pattern data. All patients who met the inclusion criteria but not exclusion criteria were included in the analysis.

### Data collection

Variables included demographic data, past medical history, use of antiplatelet or anticoagulant drugs, clinical and biological findings on admission, and treatment regimens. We categorized DAH depending on the use of AT following the classification in a previous study; DAH-AT was defined as DAH that had occurred during AT and DAH-NAT as DAH that had occurred without AT [9]. Then, as in the previous study, we classified DAH-AT depending on the presence of other causes of DAH; simple DAH-AT was defined as DAH-AT with no other causes, and complicated DAH-AT as DAH-AT with etiologies other than AT [9]. Infection was confirmed when the specific microorganism was identified by culture or serological test. Considering serological findings, radiological findings and clinical courses, we clinically diagnosed immune-related disease, drug-induced pneumonitis, heart failure, and other diseases as causes of DAH. Idiopathic DAH was defined as no detection of etiological disease of DAH.

The primary outcome was in-hospital mortality. The secondary outcome was the occurrence of complications accompanied by discontinuation of AT.

### Statistical analysis

The patients’ backgrounds and clinical characteristics on admission were compared between the survival and in-hospital death groups. These data were described as medians and interquartile ranges for quantitative variables and as counts and percentages for qualitative variables. We conducted the Mann–Whitney U test to compare continuous valuables, and Fisher's exact test to compare the proportions of categorical variables between the groups. Univariate logistic regressions were performed for the following two types of selected variables: 1) variables with p-values < 0.10 in the comparisons between the in-hospital death and survival groups, and 2) arbitrarily pre-defined variables that had already been proven as prognostic factors in the multivariate analyses in previous studies [2, 3, 5, 9]. The results were described as odds ratios (ORs) and 95% confidence intervals (CIs). We defined 60 mL/min and twice the upper limit of normal as the cutoff values of the estimated glomerular filtration rate (eGFR) and lactate dehydrogenase, respectively, as used in a previous study [2]. For the other variables, the cutoff values were determined as those that maximized the sensitivity plus specificity total in the receiver operating characteristic curve (ROC). At the same time, the area under the curve (AUC) for each ROC was calculated. Then, we compared in-hospital mortality between four groups divided according to neutrophils and lymphocytes percentages in BALF; patients with high neutrophils and lymphocytes, patients with high neutrophils and low lymphocytes, patients with low neutrophils and high lymphocytes, and patients with low neutrophils and lymphocytes. The cutoff values of neutrophils and lymphocytes percentage in BALF were same as above. We analyzed with Fisher's exact test and Bonferroni correction. Finally, we compared in-hospital mortality and the BALF cell pattern between DAH-AT and DAH-NAT, and then between complicated DAH-AT and simple DAH-AT. The threshold for significance was p < 0.05. All statistical analyses were performed in EZR software (Saitama Medical Center, Jichi Medical University, Saitama, Japan), which is a graphical user interface for R (The R Foundation for Statistical Computing, Vienna, Austria) [11].

## Results

### Patients characteristics

During the study period, 147 patients were suspected of having DAH. However, 79 patients were excluded for the following reasons: bronchoscopy was not performed (n = 59), hemorrhage from bronchial origin was responsible for the symptoms (n = 3), BALF findings were not compatible with DAH (n = 2), and the data of the BALF cell pattern were not obtained (n = 15). Consequently, 68 patients were included in our analysis (Fig. 1). The backgrounds of patients are shown in Table 1. The median age was 75 (72–80) years, and 49 (72%) patients were male. Forty-eight (71%) patients were receiving AT; 7 (10%) received antiplatelet therapy alone, 23 (34%) received anticoagulant therapy alone, and 18 (26%) received both antiplatelet and anticoagulant therapies. The etiological diseases of DAH were immune disease (8.8%), infection (13%), drug-induced pneumonitis (5.9%), heart failure (4.4%), other disease (13%), and idiopathic (54%). No variable was significantly different between the survival and death groups. The clinical characteristics of patients on admission are displayed in Table 2. Forty-seven (69%) patients received invasive or noninvasive mechanical ventilation. Fifty-six (82%) patients were administered corticosteroids. Neutrophils and lymphocytes percentages in BALF, and eGFR were significantly different between the survival and death groups.

**Table 1:** Backgrounds of patients

Variables	All patients	Survival	Death	p-value
Subject, n	68	50	18	
**Demographics**				
Age, years	75 (72–80)	75 (71–80)	76 (74–80)	0.42
Males, n	49 (72%)	37 (74%)	12 (67%)	0.56
**Comorbidities**				
Cardiac disease, n	49 (72%)	34 (68%)	15 (83%)	0.36
Respiratory disease, n	16 (24%)	12 (24%)	4 (22%)	1.00
Renal failure, n	4 (5.9%)	2 (4.0%)	2 (11%)	0.28
Malignancy, n	9 (13%)	6 (12%)	3 (17%)	0.69
**Antithrombotic therapies**				
Antithrombotic therapy, n	48 (71%)	33 (66%)	15 (83%)	0.23
Antiplatelet drugs alone, n	7 (10%)	4 (8.0%)	3 (17%)	0.37
Anticoagulant drugs alone, n	23 (34%)	16 (32%)	7 (39%)	0.77
Antiplatelet and anticoagulant drugs, n	18 (26%)	13 (26%)	5 (28%)	1.00
**Etiological diseases of DAH**				
Immune disease, n	6 (8.8%)	3 (6.0%)	3 (17%)	0.33
Drug-induced pneumonitis, n	4 (5.9%)	3 (6.0%)	1 (5.6%)	1.00
Heart failure, n	3 (4.4%)	1 (2.0%)	2 (11%)	0.17
Infection, n	9 (13%)	5 (10%)	4 (22%)	0.23
Other disease, n	9 (13%)	7 (14%)	2 (11%)	1.00
Idiopathic, n	37 (54%)	31 (62%)	6 (33%)	0.053

Data presented as median (IQR) or absolute values (percentage)

*DAH* diffuse alveolar hemorrhage, *IQR* interquartile range

**Table 2:** Clinical characteristics on admission

Variables	All patients	Survival	Death	p-value
Subject, n	68	50	18	
**Respiratory conditions**				
Oxygen flow, L/min	3 (2–10)	3 (2–10)	5 (3–10)	0.12
**Serological findings**				
White blood cell count, × 103 /μL	8.2 (6.6–10.5)	8.1 (6.6–10.4)	9.6 (6.4–10.5)	0.70
Neutrophils count, × 103 /μL (n = 66)	6.78 (4.53–8.65)	6.49 (4.49–8.80)	7.56 (5.37–8.54)	0.68
Lymphocytes, count, × 103 /μL (n = 66)	0.94 (0.69–1.25)	1.03 (0.76–1.25)	0.66 (0.43–1.35)	0.11
Hemoglobin, g/dL	10.8 (9.8–12.5)	10.7 (9.8–12.5)	11.2 (9.7–12.5)	0.95
Platelet count, × 103 /μL	198 (132–267)	206 (157–258)	179 (88.8–328)	0.68
LDH, IU/L	292 (243–379)	280 (238–363)	345 (269–388)	0.21
BUN, mg/dL	21.6 (16.1–32.7)	20.2 (15.9–30.6)	25.2 (18.5–34.9)	0.12
eGFR, mL/min	51.2 (40.8–67.8)	60.6 (43.4–71.9)	43.2 (29.8–50.9)	< 0.05
CRP, mg/dL	8.63 (3.82–13.5)	8.54 (3.48–13.2)	10.3 (7.77–14.2)	0.25
**BALF findings**				
Total cell counts in BALF, × 104 /μL	5.2 (3.1–9.4)	5.1 (3.1–8.8)	5.4 (3.9–10.8)	0.79
Neutrophils percentage in BALF, %	32.0 (13.1–47.0)	23.5 (7.35–38.8)	58.8 (41.9–70.4)	< 0.05
Lymphocytes percentage in BALF, %	19.5 (8.9–39.9)	25.3 (13.0–50.6)	8.75 (6.1–12.8)	< 0.05
**Clinical courses**				
Time from onset to admission, days	4 (2–7)	4 (2–9)	4 (2–5)	0.68
Time from admission to diagnosis of DAH, days	1 (0–3)	1 (0–3)	1 (0–3)	0.95
**Treatments**				
Invasive ventilation, n	38 (56%)	22 (44%)	16 (89%)	< 0.05
Non-invasive ventilation, n	9 (13%)	8 (16%)	1 (5.6%)	0.23
Administration of antibiotics, n	57 (84%)	39 (78%)	18 (100%)	< 0.05
Administration of corticosteroids, n	56 (82%)	40 (80%)	16 (89%)	0.49

Data presented as median (IQR) or absolute values (percentage)

*AT* antithrombotic therapy, *BALF* bronchoalveolar lavage fluid, *BUN* blood urea nitrogen, *Cr* Creatinine, *CRP* C-reactive protein, *DAH* diffuse alveolar hemorrhage, *eGFR* estimated glomerular filtration rate, *IQR* interquartile range, *LDH* lactate dehydrogenase

### Prognostic factors of in-hospital mortality

Eighteen (26.5%) patients died due to DAH in our hospital. The cutoff values for oxygen flow (4 L/min) and neutrophils (44.5%) and lymphocytes percentages (14%) in BAL were determined by the ROC curves (Additional file 2: Fig. S1). Univariate analysis of factors associated with in-hospital death are presented in Table 3. The variables significantly associated with in-hospital death were neutrophils percentage in BALF ≥ 44.5% (OR 11.4, 95% CI 3.38–38.5), lymphocytes percentage in BALF < 14% (OR 6.37, 95% CI 1.97–20.6), idiopathic DAH (OR 0.31, 95%CI 0.10–0.95), oxygen flow ≥ 4L/min (OR 3.90, 95%CI 1.20–12.6), and eGFR < 60 mL/min (OR 5.00, 95%CI 1.29–19.4). In-hospital mortality was significantly higher in patients with neutrophils percentage ≥ 44.5% and lymphocytes percentage < 14% compared with patients with neutrophils percentage < 44.5% and lymphocytes percentage ≥ 14% (Additional file 1: Table S1).

**Table 3:** Univariate analysis of variables associated with in-hospital death

Variables	Odds ratio	95% CI	p-value
Pre-existing cardiac disease	2.35	0.60–9.30	0.22
Idiopathic DAH	0.31	0.10–0.95	< 0.05
Oxygen flow ≥ 4 L/min	3.90	1.20–12.6	< 0.05
LDH > 2ULN	1.12	0.20–6.39	0.89
eGFR < 60 mL/min	5.00	1.29–19.4	< 0.05
Neutrophils percentage in BALF ≥ 44.5%	16.0	4.33–58.9	< 0.05
Lymphocytes percentage in BALF < 14%	7.44	2.11–26.2	< 0.05

*BALF* bronchoalveolar lavage fluid, *CI* confidence interval, *DAH* diffuse alveolar hemorrhage, *eGFR* estimated glomerular filtration rate, *LDH* lactate dehydrogenase, *ULN* upper limit of normal

### Prognostic influence of AT

**Fig. 2:**
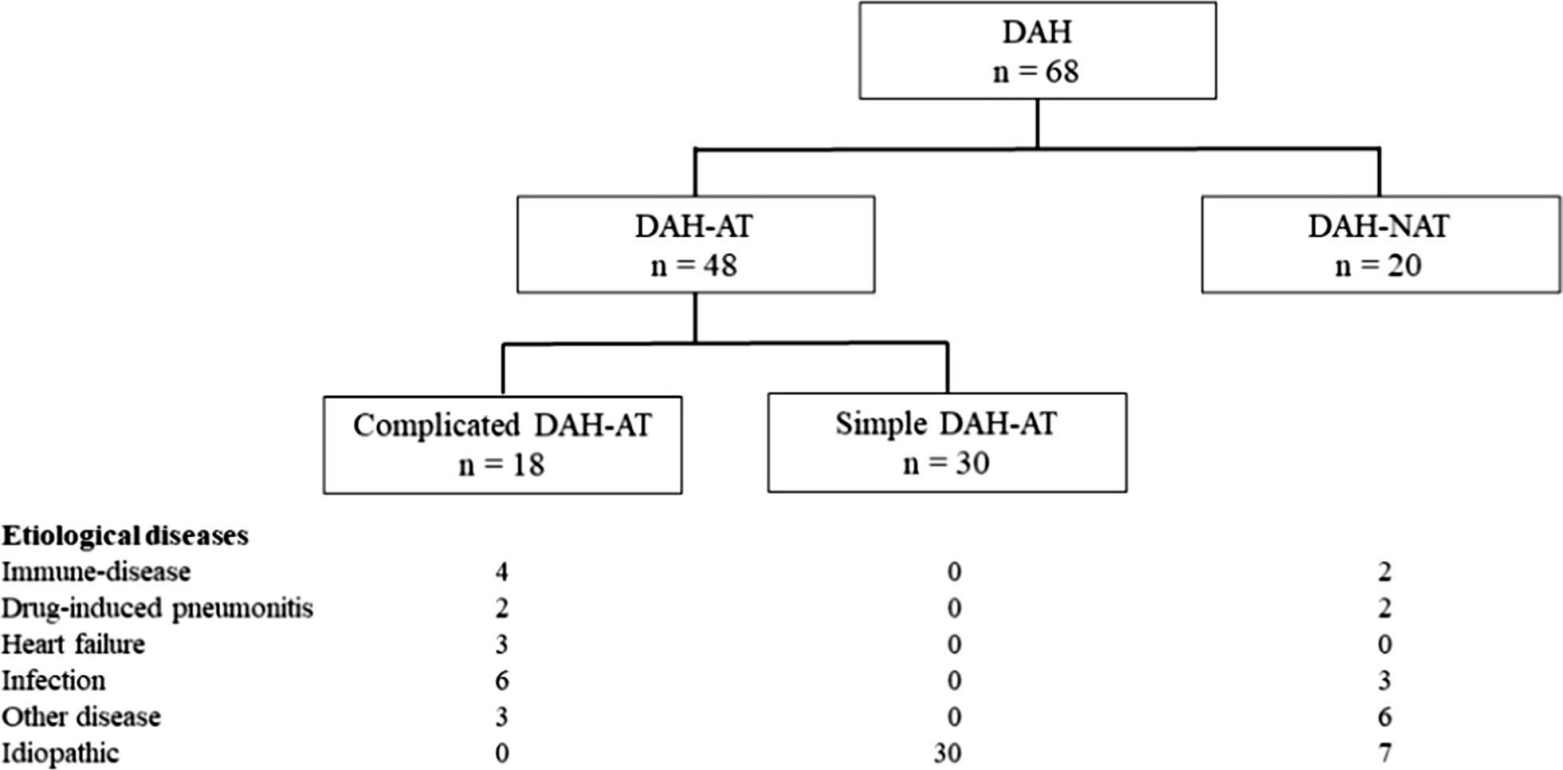
Etiological diseases of complicated DAH-AT, simple DAH-AT, and DAH-NAT. Complicated DAH-AT means DAH that occurred during AT and had causes other than AT. Simple DAH-AT means DAH that occurred during AT and had no other causes. DAH-NAT means DAH that occurred with no AT. *DAH* diffuse alveolar hemorrhage, *AT* antithrombotic therapy

Figure 2 shows the numbers of patients and their etiological diseases of complicated DAH-AT, simple DAH, and DAH-NAT. In-hospital mortality was not significantly different between DAH-AT and DAH-NAT (Additional file 1: Table S2). However, showed in-hospital mortality was significantly worse in complicated DAH-AT compared with simple DAH-AT (Additional file 1: Table S3). Regarding the BALF cell pattern, the percentage of neutrophils was significantly higher in complicated DAH-AT than in simple DAH-AT.

### Complication

An 80-year-old woman experienced stroke as a complication following discontinuation of AT. She had been administered aspirin for angina pectoris and warfarin for atrial fibrillation. She discontinued these medicines on the day of admission, and then was diagnosed as having experienced an insular cortex stroke 15 days after discontinuation, which caused higher brain dysfunction, but was not critical.

## Discussion

This study investigated the prognostic factors of DAH. The main findings were 1) High neutrophils percentage in BALF was associated with a higher rate of in-hospital deaths, and 2) A higher percentage of lymphocytes in BALF was related with lower mortality.

Table 4 lists the review of the previous observational studies of prognostic factors of DAH. In all studies, variables of respiratory conditions, such as the partial pressure of arterial oxygen/fraction of inspiratory oxygen ratio and oxygen flow, were common factors related to mortality [2, 3, 5, 9]. Furthermore, both pre-existing cardiac disease and severity of multiple organ failure were identified as factors associated with mortality in three studies [2, 3, 5, 9]. Although the influence of serological findings was analyzed in all the studies, the results differed from study to study.

**Table 4:** Review of our and previous retrospective studies of prognostic factors in DAH

Authors (year)	Prost [2]	Rabe [3]	Otoshi [9]	Mirouse [5]	Ours
**Participants of studies**					
Patients number, n	97	37	76	104	68
Patients with AT, n	Not described	Not described	39	Not described	48
IMV, n (%)	17 (18%)	32 (86%)	Not described	52 (50%)	38 (56%)
Mortality rate, n (%)	24 (25%)	19 (51%)	29 (38%)	16 (15%)	18 (27%)
*Factors associated with mortality*					
**Univariate analyses**					
Demographics	Age	–	–	Age	–
Comorbidities	CVD	–	Cardiac failure	Cardiac failure	–
AT	–	–	Complicated DAH-AT ^a^	–	Complicated DAH-AT ^a^
Etiologies	–	Classical DAH ^b^	–	APS	Idiopathic DAH
Clinical findings	Shock	Oxygen index, MODS	P/F ratio	SAPS II, Oxygen flow, P/F ratio	Oxygen flow
Serum findings	Hb, LDH, eGFR	–	–	Lymphocytes	eGFR
BALF findings	–	–	–	–	Neutrophils, Lymphocytes
Others	Smoking (> 20 pack-years)	–	–	–	–
**Multivariate analyses**					
	Shock, LDH, eGFR	Classical DAH	P/F ratio, Simple, DAH-AT	Cardiac failure, APS, SAPS II, Oxygen flow	–

*AT* antithrombotic therapy, *APS* Antiphospholipid syndrome, *BALF* bronchoalveolar lavage fluid, *CVD* cardiovascular disease, *DAH* diffuse alveolar hemorrhage, *eGFR* estimated glomerular filtration rate, *Hb* hemoglobin, *IMV* invasive mechanical ventilation, *LDH* lactate dehydrogenase, *P/F* partial pressure of arterial oxygen/fraction of inspiratory oxygen ratio, *SAPS II* Simplified Acute Physiology Score II

^a^DAH occurred during antithrombotic therapy and had causes other than antithrombotic therapy

^b^DAH associated with pulmonary vasculitis

To our knowledge, our study is the only one to have focused on the relationship between the BALF cell pattern and prognosis. We found that a high neutrophils percentage in BALF was predictive of a higher rate of in-hospital death. Increased neutrophils in BALF may be associated with neutrophil extracellular traps (NETs). Recent studies have revealed that NETs, which are released by neutrophils and composed of deoxyribonucleic acid, histones and granule-derived proteins, are cytotoxic to lung epithelium and endothelium, and can be harmful in various respiratory diseases [12, 13]. In fact, NETs concentrations in plasma and BALF have been correlated with acute respiratory distress syndrome (ARDS) severity [14, 15]. In addition, elevated serum levels of NETs have been associated with mortality in pneumonia [16]. The proportion of neutrophils reportedly was found to be related to the NETs concentration in BALF in ARDS patients [13]. Diffuse alveolar damage, which is the pathological finding in ARDS and one of the pathological patterns of DAH, has been reported to be associated with an increased percentage of neutrophils in BALF and with poor prognosis [17, 18]. These facts may result from NETs production. In DAH, as well, an increase in neutrophils in BALF may reflect an elevation of NETs in the lung, which causes acceleration of lung injury. Because lung biopsy is usually invasive and risky in the acute phase of DAH, histopathological examination of NETs is practically difficult. Instead, the BALF cell pattern may be a useful predictor of prognosis.

A high lymphocytes percentage in BALF has been found to be associated with in-hospital survival. Glucocorticoids exert a wide range of immunosuppressive activity, including the induction of T lymphocyte apoptosis [19]. The lymphocytic cell pattern in BALF was confirmed in organizing pneumonia, nonspecific interstitial pneumonia, and hypersensitivity pneumonitis, among other conditions [20]. These diseases are usually responsive to glucocorticoids and administration of glucocorticoids was recommended [21]. Additionally, a high lymphocytes percentage in BALF was correlated with good prognosis in interstitial pneumonia or acute respiratory failure in the studies that most of cases had been administered glucocorticoids [22, 23]. We speculate that enriched lymphocytes in BALF is associated with good responsiveness to glucocorticoids and, as a result, with good prognosis in DAH. However, our analysis showed that in patients with higher neutrophils percentage in BALF, higher lymphocytes did not correlate with lower in-hospital mortality. Coexisting with neutrophilic inflammation, glucocorticoids treatment may not indicate a significant effect in spite of enriched lymphocytes.

In our study, lower eGFR was associated with higher in-hospital mortality, as also found in a previous study [2]. Renal dysfunction has been proven as a prognostic factor in various systemic diseases listed as etiologies of DAH in our study, such as anti-neutrophil cytoplasmic antibody-related vasculitis, chronic heart failure and community-acquired pneumonia [24–26]. Low eGFR as prognostic factor in our study might reflect those underlying diseases.

In the present study, AT was not significantly correlated with prognosis. However, in-hospital mortality was significantly higher in complicated DAH-AT than in simple DAH-AT. Patients with complicated DAH-AT tended to have worse mortality because of systemic disease and that is why they tended also to have neutrophilic inflammation in their BALF.

Our study findings suggest a trivial risk of complications of discontinuation of AT in DAH. The occurrence of complications was not significantly different between discontinuation and continuation. Therefore, physicians should not hesitate to discontinue AT in DAH, if necessary.

There were two limitations in our study. First, this was a retrospective single-center study with a small number of patients. Our sample size was insufficient for multivariate analysis. Second, our analysis excluded 59 patients who had not received bronchoscopy, so we might have missed patients who were so severe that we could not perform BAL.

## Conclusions

In conclusion, a higher neutrophils percentage in BALF was found to be significantly associated with in-hospital mortality. On the other hand, a higher lymphocytes percentage in BALF was correlated with lower mortality.

## Supplementary Information

**Additional file 1. Supplemental Table 1:** Comparison of corticosteroid use and in-hospital mortality among four groups according to neutrophils and lymphocytes percentages in BALF. **Supplemental Table 2:** Comparison of the BALF cell pattern and in-hospital mortality between DAH-AT and DAHNAT. **Supplemental Table 3:** Comparison of the BALF cell pattern and in-hospital mortality between complicated DAH-AT and simple DAH-AT.

**Additional file 2. Fig. S1**: Receiver operating characteristic curves for predicting in-hospital death for the factors oxygen flow (A), neutrophils percentage (B), and lymphocytes percentage (C) in bronchoalveolar lavage fluid. AUC: area under curve; BALF: bronchoalveolar lavage fluid.

## Data Availability

The datasets used and/or analyzed during the current study are available from the corresponding author on reasonable request.

## Abbreviations

ARDS: Acute respiratory distress syndrome
AT: Antithrombotic therapy
AUC: Area under the curve
BAL: Bronchoalveolar lavage
BALF: Bronchoalveolar lavage fluid
Cl: Confidence interval
DAD: Diffuse alveolar damage
DAH: Diffuse alveolar hemorrhage
DAH-AT: Diffuse alveolar hemorrhage that occurred during antithrombotic therapy
DAH-NAT: Diffuse alveolar hemorrhage that occurred without antithrombotic therapy
eGFR: Estimated glomerular filtration rate
NETs: Neutrophil extracellular traps
ORs: Odds ratios
ROC: Receiver operating characteristic curve
