# Supplementary material for: High neutrophils and low lymphocytes percentages in bronchoalveolar lavage fluid are prognostic factors of higher in-hospital mortality in diffuse alveolar hemorrhage
Source: BMC Pulm Med. 2021 Sep 9;21:288. doi: 10.1186/s12890-021-01660-x (PMC8431931; doi:10.1186/s12890-021-01660-x)
Supplement: Supplementary file 1 — Additional file 1. Supplemental Table 1: Comparison of corticosteroid use and in-hospital mortality among four groups according to neutrophils and lymphocytes percentages in BALF. Supplemental Table 2: Comparison of the BALF cell pattern and in-hospital mortality between DAH-AT and DAHNAT. Supplemental Table 3: Comparison of the BALF cell pattern and in-hospital mortality between complicated DAH-AT and simple DAH-AT. [file 12890_2021_1660_MOESM1_ESM.docx]

Supplemental Table 1 Comparison of corticosteroid use and in-hospital mortality among four groups according to neutrophils and lymphocytes percentages in BALF

| Neutrophils percentage in BALF | High (> 44.5%) | | Low (< 44.5%) | |  |
| --- | --- | --- | --- | --- | --- |
| Lymphocytes percentage in BALF | High (> 14%) | Low (<14%) | High (> 14%) | Low (<14%) | p-value |
| Patients, n | 6 | 14 | 32 | 16 |  |
| corticosteroids use, n (%) | 3 (50%) | 11 (79%) | 28 (88%) | 14 (88%) | 0.17 |
| In-hospital mortality, n (%) | 3 (50%) | 10 (71%) | 1 (3.1%) | 4 (25%) | <0.05 ^a^ |
| BALF bronchoalveolar lavage fluid  ^a^ < 0.05 for patients with high neutrophils and low lymphocytes vs patients with low neutrophils and high lymphocytes in bonferroni correction | | | | | |

Supplemental Table 2 Comparison of the BALF cell pattern and in-hospital mortality between DAH-AT and DAH-NAT

| Variables | DAH-AT ^a^ | DAH-NAT ^b^ | p-value |
| --- | --- | --- | --- |
| Patients, n | 48 | 20 |  |
| Neutrophils percentage in BALF, % | 33.3 (19.4-48.3) | 31.0 (6.98-43.8) | 0.5 |
| Lymphocytes percentage in BALF, % | 13.3 (8.38-28.0) | 41.0 (20.8-53.0) | <0.05 |
| In-hospital mortality, n (%) | 15 (31%) | 3 (15%) | 0.23 |
| Data presented as median (IQR) or absolute values (percentage).  ^a^ DAH that occurred during antithrombotic therapy  ^b^ DAH that occurred without antithrombotic therapy  AT antithrombotic therapy, BALF bronchoalveolar lavage fluid, DAH diffuse alveolar hemorrhage, IQR interquartile range. | | | |

Supplemental Table 3 Comparison of the BALF cell pattern and in-hospital mortality between complicated DAH-AT and simple DAH-AT

| Variables | complicated DAH-AT ^a^ | simple DAH-AT ^b^ | p-value |
| --- | --- | --- | --- |
| Patients, n | 18 | 30 |  |
| Neutrophils percentage in BALF, % | 55.8 (29.6-69.6) | 24.8 (11.4-41.4) | <0.05 |
| Lymphocytes percentage in BALF, % | 13.3 (8.00-22.1) | 13.3 (8.63-33.8) | 0.64 |
| In-hospital mortality, n (%) | 10 (56%) | 5 (17%) | <0.05 |
| Data presented as median (IQR) or absolute values (percentage).  ^a^ DAH that occurred during antithrombotic therapy and had causes other than antithrombotic therapy  ^b^ DAH that occurred during antithrombotic therapy and had no other cause  AT antithrombotic therapy, BALF bronchoalveolar lavage fluid, DAH diffuse alveolar hemorrhage, IQR interquartile range. | | | |
