# Supplementary figures and images for: High neutrophils and low lymphocytes percentages in bronchoalveolar lavage fluid are prognostic factors of higher in-hospital mortality in diffuse alveolar hemorrhage
Source: BMC Pulm Med. 2021 Sep 9;21:288. doi: 10.1186/s12890-021-01660-x (PMC8431931; doi:10.1186/s12890-021-01660-x)

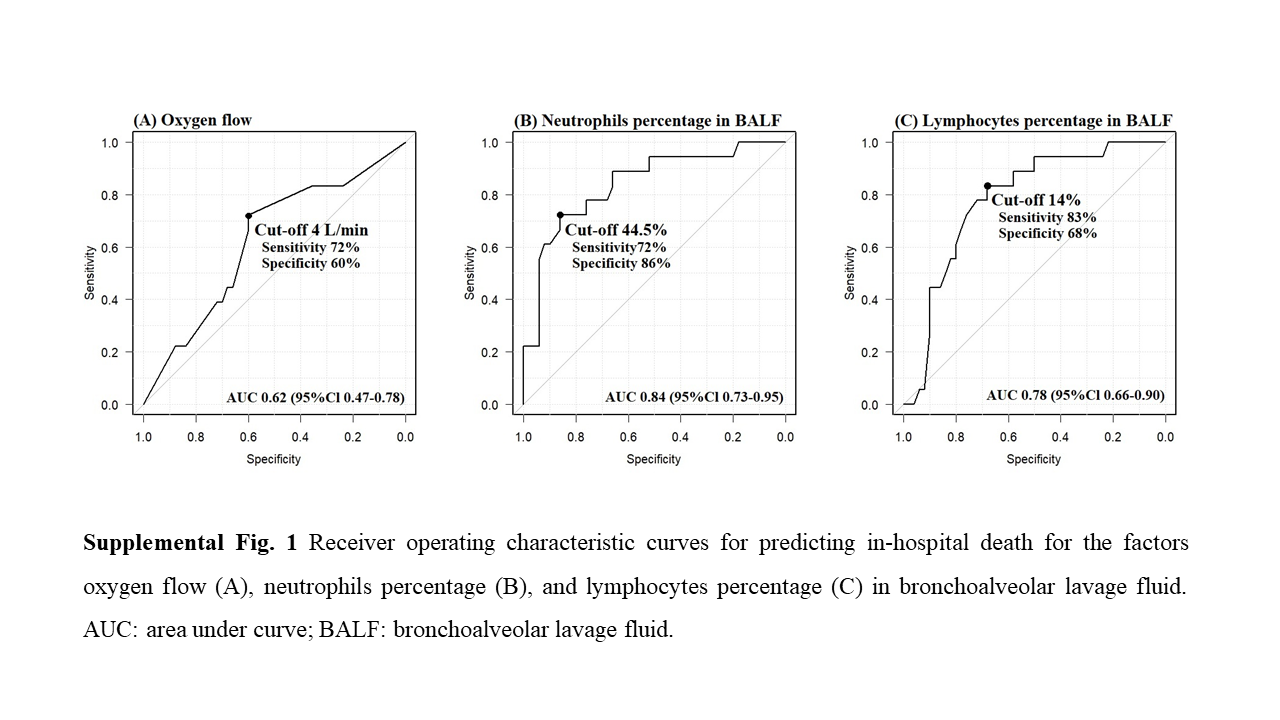

Supplement: Supplementary file 2 — Additional file 2. Fig. S1: Receiver operating characteristic curves for predicting in-hospital death for the factors oxygen flow (A), neutrophils percentage (B), and lymphocytes percentage (C) in bronchoalveolar lavage fluid. AUC: area under curve; BALF: bronchoalveolar lavage fluid. [file 12890_2021_1660_MOESM2_ESM.png]
